# Supplementary material for: BMP9-ID1 Pathway Attenuates N6-Methyladenosine Levels of CyclinD1 to Promote Cell Proliferation in Hepatocellular Carcinoma
Source: Int J Mol Sci. 2024 Jan 12;25(2):981. doi: 10.3390/ijms25020981 (PMC10816017; doi:10.3390/ijms25020981)
Supplement: Supplementary file 1 [file ijms-25-00981-s001.zip › Figure S1.pdf]

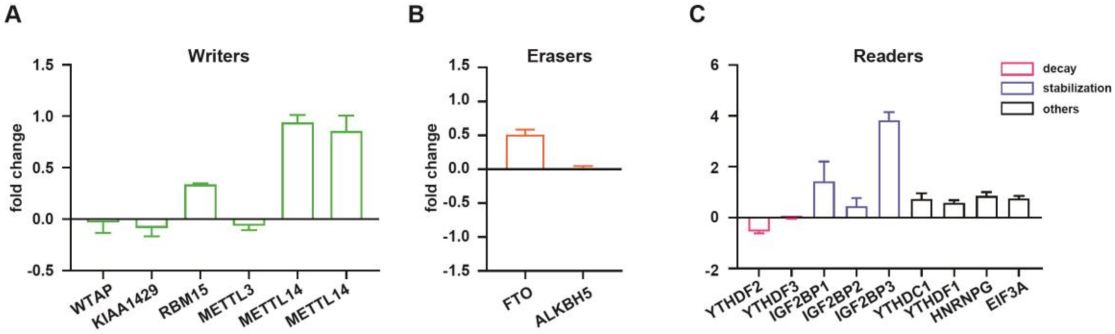

Figure S1. The effect of BMP9 on m6A writers, erasers and readers. (A) Relative gene expression levels of m6A writers in Huh7. (B) Relative gene expression levels of m6A erasers in Huh7. (C) Relative gene expression levels of m6A readers in Huh7. Cells were treated with DMSO or BMP9 (5 ng/mL) for 48 h. The error bars represent the SD from at least three independent biological replicates.
