# Supplementary material for: BMP9-ID1 Pathway Attenuates N6-Methyladenosine Levels of CyclinD1 to Promote Cell Proliferation in Hepatocellular Carcinoma
Source: Int J Mol Sci. 2024 Jan 12;25(2):981. doi: 10.3390/ijms25020981 (PMC10816017; doi:10.3390/ijms25020981)
Supplement: Supplementary file 1 [file ijms-25-00981-s001.zip › Table S2.pdf]

Supplementary Table S2. Primers Used for RT-qPCR.

Primer Name	Sequence (5’-3’)
β-actin Forward	CTACCTCATGAAGATCCTCACCGA
β-actin Reverse	TTCTCCTTAATGTCACGCACGATT
ID1 Forward	GTTGGAGCTGAACTCGGAATCC
ID1 Reverse	ACACAAGATGCGATCGTCCGCA
CyclinD1 Forward	CGATGCCAACCTCCTCAACGA
CyclinD1 Reverse	TCGCAGACCTCCAGCATCCA
CCND1-5'UTR Forward	CAGTAACGTCACACGGACTAC
CCND1-5'UTR Reverse	GTCTGTAGCTCTCTGCTACTGCG
FTO Forward	CCCTGTGAGCAGCAACATAAG
FTO Reverse	CAACCCGACCCAGTCTAAATC
YTHDF2 Forward	ATAGGAAAAGCCAATGGAGGG
YTHDF2 Reverse	CCAAAAGGTCAAGGAAACAAAG
